# Evaluation of filter substrates for concurrent optical photothermal infrared and Raman spectroscopy in microplastic analysis

**DOI:** 10.1007/s00216-026-06735-4

**Published:** 2026-08-25

**Authors:** Shiwani Shiwani, Shravan Raghunathan, Jürgen Popp, Iwan W. Schie, Christoph Krafft

**Affiliations:** 1 Leibniz Institute of Photonic Technology, Albert-Einstein-Straße 9, 07745 Jena, Germany; 2 Institute of Physical Chemistry and Abbe Center of Photonics, Friedrich Schiller University Jena, Helmholtzweg 4, 07743 Jena, Germany; 3 Department for Medical Engineering and Biotechnology, University of Applied Sciences Jena, Carl-Zeiss-Promenade 2, 07745 Jena, Germany

**Keywords:** IR spectroscopy/Raman spectroscopy, Sampling, Polymers

## Abstract

Optical photothermal infrared (O-PTIR) spectroscopy is an emerging technique to analyze submicron mid-infrared absorption. It can be combined with concurrent, co-located Raman spectroscopy on the same instrument platform, offering considerable potential for microplastic analysis. However, since measurements are typically performed directly on collection filters, substrate selection becomes a critical yet un-examined factor for these concurrent modalities. Here, we characterized nine commercially available filter substrates, namely Anodisc, silicon, gold-coated PET, gold-coated PC, silver, glass and quartz microfiber, PC, and cellulose, for O-PTIR and Raman analysis using 14 µm and 7 µm PMMA microplastic beads. Spectral fidelity was quantified via hit quality indices against a CaF_2_ reference. Our results show that substrate suitability is governed by modality-specific requirements. O-PTIR demands mid-infrared spectral neutrality and is additionally sensitive to the substrate surface, whereas the quality of Raman spectra primarily depends on the absence of substrate Raman bands. Beyond spectral match quality, the reproducibility of PMMA spectra is consistently higher in the Raman channel than in the O-PTIR channel. This difference is attributed to O-PTIR’s sensitivity to local thermal contact and surface morphology, which introduce measurement variability absent in direct Raman scattering. Of the tested substrates, Anodisc and gold-coated PET performed the best in both modalities and with both particle sizes. These findings demonstrate that recommendations for substrates established for conventional FTIR or Raman microscopy cannot be transferred directly to O-PTIR. They also provide an evidence-based framework for selecting substrates in multimodal microplastic analysis workflows.

**Supplementary Information:**

The online version contains supplementary material available at https://doi.org/10.1007/s00216-026-06735-4.

## Introduction

Global plastic production has grown from ~ 2 million tons in the early 1950 s to roughly 464 million tons by 2018 [1, 2]. Because only about 9% is recycled, physical fragmentation and chemical degradation of discarded plastics generate particles known as microplastics, defined as plastic fragments roughly 1 µm to 5 mm in size [3]. Inhaling or ingesting microplastic particles may pose a risk to human health [4, 5]. To assess this potential risk, analytical methods must first identify the polymer type of individual microparticles. This is a critical first step toward tracing sources and estimating exposure. When inhaled or ingested, microplastics can transport adsorbed contaminants, posing risks to human health [6–8]. Characterizing this contamination requires analytical methods that identify the polymer type of individual microparticles, a critical first step toward tracing sources and assessing risks. Two analytical routes currently exist: destructive thermo-analytical techniques and non-destructive vibrational spectroscopic methods. Pyrolysis gas chromatography mass spectrometry (py-GC/MS) [9] provides mass-quantitative polymer profiles but irreversibly consumes each analyzed particle, precluding determination of particle counts, sizes, or shapes. These constraints have accelerated the shift toward vibrational techniques, which preserve individual particles intact while providing chemically specific information. While imaging-based methods do not directly yield mass concentrations, these can be approximated from particle volume and density [10].

To overcome the loss of particle information inherent to thermo-analytical methods, researchers increasingly rely on micro-Fourier transform infrared (micro-FTIR), quantum cascade laser (QCL) infrared and micro-Raman spectroscopy, which detects particles on a substrate [11–14]. Extracting those particles from complex matrices entails a multistage workflow. Environmental samples are first coarsely sieved to remove macro debris, and then subjected to density separation to float low density polymers away from mineral grains and finally exposed to enzymatic or chemical digestion to dissolve residual organic matter without damaging most plastic particles [15]. The cleaned suspension is then vacuum filtered onto microporous filter membranes with different pore sizes, ensuring retention of microplastics. Particles captured on these filters are identified in situ by vibrational spectroscopy, a practice that minimizes handling and thus reduces contamination risk. Yet, this very convenience introduces a hidden variable, the filter substrate itself. Infrared (IR) absorption or Raman scattering originating from the substrate can mask or distort polymer fingerprints, yielding ambiguous classifications and biased particle counts. Consequently, selecting a substrate is an essential though often underestimated precondition for reliable microplastic analysis [16].

Across optical systems, the notion of a “spectroscopically silent” filter turns out to be highly method specific. In transmission micro-FTIR imaging, conventional anodized aluminum (Anodisc) filters are transparent only above ≈1250 cm⁻^1^, whereas a micro etched silicon wafer remains essentially flat from 4000 to 600 cm⁻^1^ [15]. QCL-IR and Raman microscopy benefit from reflective metal coatings. Vapor depositing a 100-nm aluminum layer onto a track etched polycarbonate (PC) filter produces a mirror-like substrate that adds virtually no IR and Raman bands and tolerates laser powers up to ≈3 mW [17]. In a separate airborne PM study, Wright et al. [18] screened six commercial membranes and found that only silver filters provided enough optical contrast and low background to identify 1–10 µm plastics by Raman spectral imaging; quartz filters offered insufficient visual contrast, PTFE and alumina membranes were incompatible with Raman spectral imaging mapping despite particles being optically visible, and cellulose filters burned under routine laser powers when loaded with particulate matter. Similarly, a laser direct infrared (LDIR) study found that the textured surface of silver membranes degraded both image quality and particle identification reliability, even for particles above 20 µm [19]. MirrIR slides and gold-coated filters performed adequately as reflecting substrates [19]. These substrate-dependent trade-offs are now described in practical guidelines. Schymanski et al. [20] guideline recommends silicon or gold/aluminum-coated PC membranes for both micro-FTIR and micro-Raman analysis and cautions against PTFE or cellulose filters. Sigma Aldrich’s filter membrane selection note [21] offers the same recommendation.

These substrate requirements are technique-specific because they arise from distinct detection mechanisms. In transmission FTIR, the beam passes through the substrate, so the sole requirement is mid-IR transparency; surface texture and thermal properties are irrelevant. In confocal Raman microscopy, the laser is focused into the particle and Raman-scattered photons are collected from that volume, so what matters is the absence of substrate Raman bands, not surface finish or thermal conductivity. O-PTIR spectroscopy operates differently. A pulsed mid-IR laser heats the sample, and a co-aligned visible probe detects the resulting refractive-index modulation in reflection. The substrate must therefore be simultaneously mid-IR silent, optically smooth at the probe wavelength, and thermally compatible with reproducible photothermal detection, a combination of constraints that neither FTIR nor Raman imposes. While previous comparisons were performed with micro-FTIR, QCL-IR, LDIR, and Raman, none has yet been extended to O-PTIR spectroscopy. Early case studies already highlight its potential [22]. Co-registered O-PTIR and Raman spectra were acquired from microplastics on MirrIR reflective glass slides [23], and microplastics collected from intravenous fluid sets were characterized directly on Anodisc filters [24]. Despite these promising demonstrations, no study has systematically screened commercially available filter substrates under identical O-PTIR conditions, leaving substrate-specific background effects unquantified. To close this gap, the present work benchmarks a broad panel of nine commercially available filters on a single O-PTIR platform with simultaneous Raman readout, generating the first comprehensive dataset for evidence-based substrate selection in microplastic analysis.

## Materials and methods

### Filter substrates

Commercially available filters with varying pore sizes, chemical compositions, and manufacturing sources were selected to reflect the options typically used in microplastic research. In total, nine substrates were tested encompassing different materials. Table 1 provides each filter’s manufacturer, product code, diameter, and pore size, enabling a clear overview of the substrates used.

**Table 1:** Overview of the nine filter substrates benchmarked in this study, including material composition, manufacturer, product code, filter diameter, and nominal pore size

No.	Code	Material	Manufacturer	Product code	Diameter (mm)	Pore (µm)
1	Glass microfiber	Glass	Cytiva Europe	1821-025	25	0.45
2	Quartz microfiber	Quartz	Cytiva Europe	1851-025	25	0.45
3	Gold-PET	Gold-coated PET	i3 Membrane	201–122022-5030	30	5
4	Gold-PC	Gold-coated PC	i3 Membrane	201–01010-0825	25	0.8
5	Silver	Silver	Sigma-Aldrich Chemie	AG4502550	25	0.45
6	Anodisc	Aluminum oxide	Altmann Analytic	WHA68096022	25	0.2
7	Silicon	Silicon	Smart Membranes	P12M6-250	10	5
8	PC	Polycarbonate	Cytiva Europe	7060-4713	47	5
9	Cellulose	Mixed cellulose ester	Cytiva Europe	10400812	47	1.2

### Microplastic samples

To ensure reproducible and controlled measurement conditions, polymethyl methacrylate (PMMA) microspheres with nominal diameters of 7 µm (PMMA/F-L-574) and 14 µm (PMMA/F-W53) were used. PMMA was selected because commercially available monodisperse microspheres provide well-defined particle sizes, shapes, and surface properties, allowing differences in spectral quality to be attributed primarily to the filter substrate rather than to variations in particle morphology. The particles were obtained in powdered form from Microparticles GmbH and dispersed in distilled water prior to deposition on the filter substrates. Each suspension was then pipetted onto the filter substrates and allowed to air dry under ambient conditions, resulting in the deposition of PMMA particles on the filter surfaces for subsequent O-PTIR and Raman analysis.

### O-PTIR system

Measurements were performed with mIRage-R (Photothermal Spectroscopy Corp., USA) that contains a co-located IR and Raman microscope. In O-PTIR operation, a nanosecond pulsed mid-IR pump laser thermally excites the sample, while a collinear continuous wave 785 nm probe laser monitors the resulting refractive-index modulation, delivering FTIR-quality spectra in reflection geometry at submicrometer resolution. Details were previously described [23]. The O-PTIR system is equipped with a four-module pulsed quantum cascade laser (QCL, MIRcat 2400, Daylight Solution) with a tunable range from 934 to 1800 cm^−1^ and 2700 to 2992 cm^−1^, and a 785 nm probe laser. The IR laser repetition rate was set at 100 kHz at 980 ns per pulse. The O-PTIR single spectra and single wavenumber images were acquired at 47% IR power (0.3–2.3 mW depending on wavenumber), to avoid local overheating, 21% probe power (2.88 mW), and 1000 cm^−1^ s^−1^ scan speed, using a 40 × reflective Cassegrain objective (NA 0.78) in reflection mode.

For the Raman spectra, the 785 nm probe laser could be set at ≈ 15 mW (100% power at the sample) without detectable photobleaching or filter damage. Raman spectra were acquired at an exposure time of 6 s, from 174 to 1879 cm⁻^1^ using a 600 lines mm⁻^1^ grating centered at 1027 cm⁻^1^ and a 1024 × 255-pixel deep depletion, back illuminated CCD. Six successive frames were co-added with cosmic ray correction.

### Data acquisition

Each filter was mounted on the mIRage-R microscope stage and was inspected using a 10 × VIS or 40 × IR objective in bright-field reflection mode to locate well-isolated PMMA spheres. Three well-separated beads per substrate were selected at random. After autofocusing the probe laser on the bead surface, a rapid discrete wavenumber image was then collected at 1727 cm⁻^1^, the strong prominent *ν*(C = O) band of PMMA [23], to generate a carbonyl absorbance image, that precisely defined bead position. The pixel of maximum absorbance was subsequently probed with a full range O-PTIR scan (934 → 2992 cm⁻^1^, 6.6 cm⁻^1^ resolution, four co-adds), after which a co-registered Raman spectrum was acquired at the same coordinate using the 785 nm probe (174–1879 cm⁻^1^, six co-adds). This discrete wavenumber image to spot protocol yielded three paired IR/Raman spectra for each filter substrate. In addition, three blank regions per filter were recorded under identical settings to obtain substrate-only spectral contribution. All spectra were acquired as targeted single-point measurements on manually located beads.

### Data processing

Spectra from the two channels (IR/Raman) were treated separately but evaluated with the same match metric. For the O-PTIR channel, each spectrum was first area normalized and then compared with a CaF_2_ supported PMMA reference using the cosine similarity hit quality index (HQI) [23], defined as

$$HQI=\frac{({x\cdot y)}^{2}}{{\parallel x\parallel }^{2}{\parallel y\parallel }^{2}}\times 100$$

where *x* and *y* are the area normalized absorbance vectors of the unknown and reference spectra, respectively; values range from 0 (no match) to 100 (perfect match).

Raman spectra required preprocessing before HQI calculation. Wavenumber calibration was carried out with a crystalline 4-acetaminophenol standard. The well-characterized peaks were used to align the instrument-assigned wavenumber positions via a fourth-order polynomial fit, ensuring accurate wavenumber calibration. Following wavenumber calibration, intensity calibration is conducted using a NIST standard white calibration lamp (Kaiser Optical System, USA). This step corrects for the intensity response function of the entire optical system, which includes the objective lens, filters, and CCD detector, compensating for variations in quantum efficiency across wavenumbers [23, 25]. Broad fluorescence and scattering backgrounds were removed with asymmetric least squares (AsLS) baseline correction (*λ* = 10^6^, *p* = 0.01, 10 iterations). The spectra were then smoothed with an 11-point, third-order Savitzky-Golay filter and area normalized. The resulting pre-processed Raman spectra were finally compared with a PMMA reference spectrum recorded on CaF_2_ via the same HQI metric described above.

## Results

### O-PTIR characterization of 14 µm PMMA beads

**Fig. 1:**
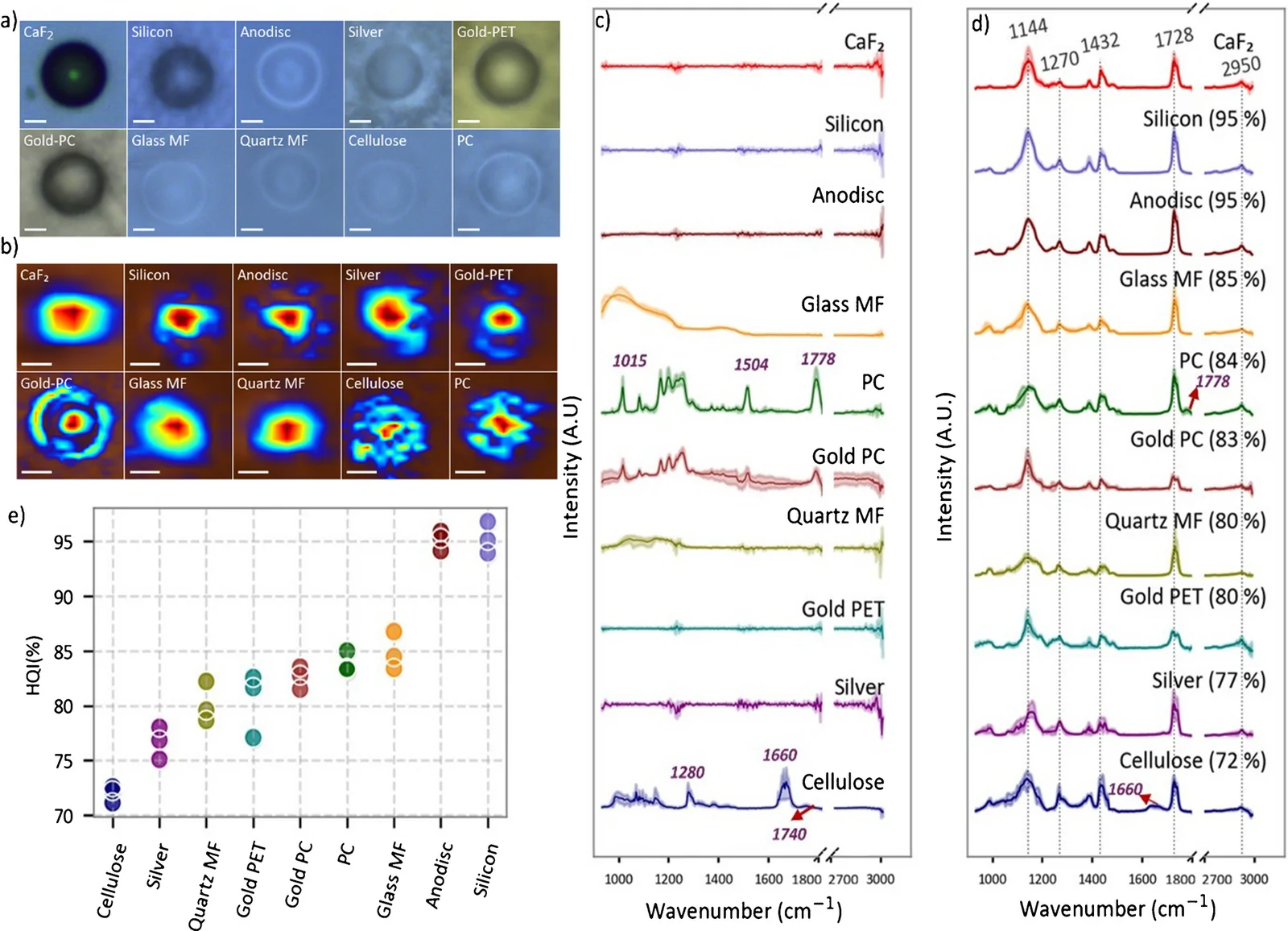
Performance of nine filter substrates for O-PTIR analysis of a 14 µm PMMA bead. **a** Bright-field images of 14 µm PMMA deposited on each substrate; CaF_2_ serves as the reference slide. Scale bar = 5 µm. **b** Single band O-PTIR discrete wavenumber images acquired at 1728 cm⁻^1^ (ν C = O of PMMA) confirm bead location prior to full spectral scans. Scale bar, 5 µm. **c** Background O-PTIR spectra of the blank substrates; key absorption bands of every substrate are labeled. Variations near 1220 cm^−1^ are due to low IR intensities at QCL transitions. **d** O-PTIR spectra of a 14 µm PMMA bead on every substrate (three beads per substrate; solid line = mean, shaded envelope =  ± 1 SD). Characteristic PMMA bands at 1144, 1270, 1432, 1728, and 2950 cm⁻^1^ are preserved on all filters. **e** HQI, cosine similarity to a CaF_2_ supported PMMA reference expressed as percentages; points are individual beads: Anodisc and silicon yield the highest match scores (~ 95%), and gold-coated and microfiber filters cluster at 80–84%, while silver and cellulose fall below 80% due to surface scattering and overlapping cellulose bands, respectively. In **c** and **d**, spectra are offset vertically for clarity

Figure 1a shows bright-field images of a single 14 µm PMMA sphere placed on each substrate, with a CaF_2_ slide used as a reference. The bead is clearly visible on the silicon substrate and the gold-coated PC/PET filter substrates. However, Anodisc, polycarbonate (PC), mixed cellulose ester (cellulose), and glass/quartz microfiber filters offer lower optical contrast and partially veil the particle, making it harder to pinpoint. A comparable challenge is encountered with the silver filter. Its coarse, sintered metal surface diffusely scatters light, blurring the edges of the bead and lowering image contrast. To confirm bead position on every substrate, Fig. 1b presents a single-band O-PTIR discrete wavenumber image at 1727 cm⁻^1^ (PMMA ν C = O); this quick chemical image ensures that the probe is precisely centered on the bead before the subsequent full range O-PTIR and Raman scans.

The discrete wavenumber images in Fig. 1b reveal different intensity patterns between the substrates. Concentric rings appear on the gold-coated PC and gold-coated PET filters. PMMA beads are transparent, so some of the probe light passes through the bead and is reflected by the underlying substrate surface. The reflected light from the bead surface interferes with this reflected light and creates the ring pattern. On the mixed cellulose ester filter, the bead appears with reduced contrast and a patchy background because the substrate itself absorbs IR radiation near the 1727 cm^−1^ imaging wavenumber (ester band at approximately 1740 cm^−1^).

To evaluate spectral overlap with polymer bands, the O-PTIR background spectra of the nine substrates and the CaF_2_ reference are shown in Fig. 1c. The glass and quartz microfiber filters both feature broad Si–O–Si stretching around 900–1200 cm⁻^1^ [26]. Polycarbonate (PC) displays a C = O stretch near 1780 cm⁻^1^ [23] and an aromatic ring vibration at 1504 cm⁻^1^ [23], along with symmetric O–C–C carbonate deformations near 1015 cm⁻^1^ [27]. Gold-coated PC preserves these PC features because its polymer base remains spectrally active beneath the metal coating. In mixed cellulose ester filters, which often contain cellulose nitrate and cellulose acetate, the 1658 cm⁻^1^ band can reflect asymmetric nitro stretching (from cellulose nitrate), while a feature near 1280 cm⁻^1^ corresponds to symmetric nitro stretching. Additional ester related absorption (e.g., from cellulose acetate) appears near 1740 cm⁻^1^ [28]. Meanwhile, silicon, Anodisc, silver, and gold-coated PET exhibit minimal absorption in the measured range, providing lower interference for microplastic analysis.

Following the background assessment, Fig. 1d presents the O-PTIR spectra obtained for a 14 µm PMMA bead on each substrate. For each substrate, spectra from three independent beads were recorded; the thicker trace represents the mean spectrum, and the surrounding shaded region represents its standard deviation. Individual spectra of all beads are shown in Supplementary Fig. S1. The spectrum of a CaF_2_ supported bead serves as the reference. In every case, the characteristic PMMA bands are retained: 1144 cm⁻^1^ (symmetric ν C–O–C stretch), 1270 cm⁻^1^ (asymmetric ν C–O–C stretch + CH₃ wag), 1432 cm⁻^1^ (CH₃/CH₂ deformation), 1728 cm⁻^1^ (ester C = O stretch), and 2950 cm⁻^1^ (asymmetric CH₃ stretch).

For qualitative assessment, each bead spectrum was matched to a PMMA reference and assigned a HQI. Mean HQIs for the three beads on every substrate are listed beside the stacked spectra in Fig. 1d, with individual values plotted in Fig. 1e. Anodisc and silicon sit at the top (HQI ≈95%); their flat backgrounds and smooth surfaces preserve the full PMMA fingerprint with minimal distortion. Gold-coated PET/PC, PC, glass microfiber, and quartz microfiber follow at HQI ≈ 80–85%, a range that still denotes reliable identification. In the gold-coated PET filter, the evaporated metal layer completely masks the underlying polymer, so no substrate bands appear across 900–3000 cm⁻^1^. Gold-coated PC, despite its metal coating, retains faint PC features in the background but still yields recognizable PMMA spectra at the 14 µm scale (HQI ≈ 83%). Filter substrate PC, by contrast, retains its intrinsic carbonyl shoulder at 1778 cm⁻^1^, but nonetheless, PMMA bands are still recognizable. The remaining substrates, silver (HQI ≈ 77%) and cellulose (HQI ≈ 72%), fall below the 80% value. The cellulose substrate is penalized by cellulose bands that overlap key PMMA bands. Though virtually IR silent, the silver filter shows reduced spectral match and larger bead-to-bead variability; its coarse sintered surface is likely the cause (individual spectra shown in Supplementary Fig. S1). This drawback echoes the LDIR study [19], in which textured silver filters likewise produced spurious spikes and frequent misidentifications. These results demonstrate that O-PTIR identification requires spectrally transparent substrates that allow clean reflection of the probe beam. Anodisc and silicon meet both conditions almost completely and therefore give higher match scores. Close behind, the gold-coated PET and PC filters, together with the glass and quartz microfiber filters, also support reliable PMMA identification (HQI ≥ 80%).

### O-PTIR characterization of 7 µm PMMA beads

**Fig. 2:**
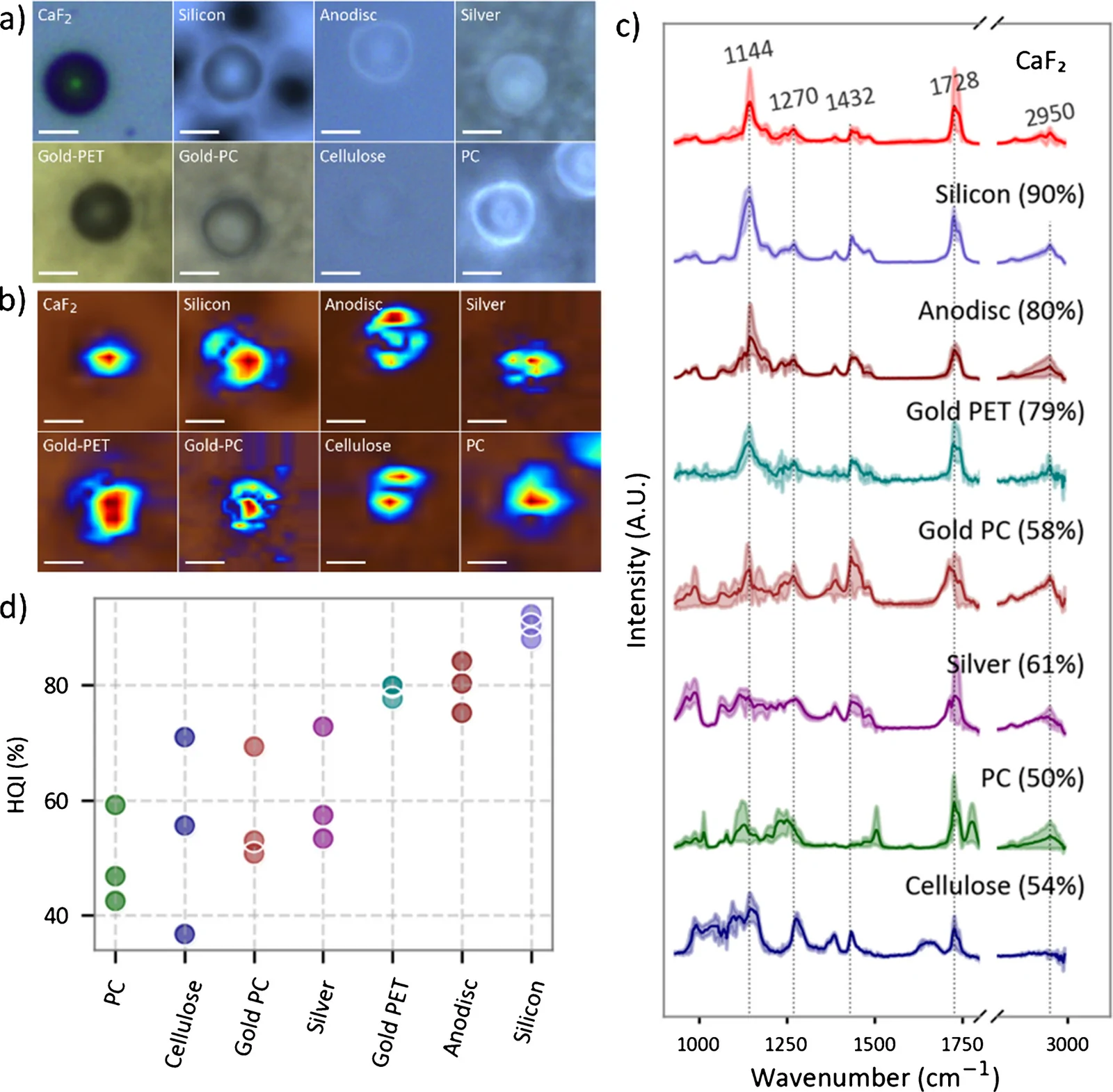
O-PTIR performance of seven filter substrates with 7 µm PMMA beads. **a** Bright-field reflection images of 7 µm PMMA sphere on each substrate. Glass and quartz microfiber filters could not provide sufficient optical contrast for bead localization and were therefore excluded from this size class. Scale bar = 5 µm. **b** Single band O-PTIR maps at 1727 cm⁻^1^ (ν C = O of PMMA) confirm the bead center on every remaining substrate. Scale bar = 5 µm. **c** Full range O-PTIR spectra collected from three independent beads per substrate (solid line = mean, shaded band =  ± 1 SD). **d** HQI versus a CaF_2_ supported PMMA reference for every bead. Mean HQIs: silicon ≈ 90%, Anodisc ≈ 80%, gold-coated PET ≈ 79%; gold-coated PC ≈ 58%, PC ≈ 50%, cellulose ≈ 54%, silver ≈ 61%. Only silicon, Anodisc, and gold-coated PET consistently exceed the ~ 70% reliability threshold for unambiguous PMMA identification at the 7 µm particle scale. In **c**, spectra are offset vertically for clarity

Figure 2a shows bright-field images of single 7 µm PMMA spheres on the seven substrates used. As in Fig. 1a, it was difficult to locate the beads on glass and quartz microfiber filters. The dense, translucent fiber mats provided insufficient contrast; therefore, those two filters were omitted for the 7 µm evaluation. The rough, sintered silver filter also offered poor optical contrast, resulting in blurring bead edges. However, careful focusing and scanning still reveals the microplastic bead. To collect spectra exactly at the center of the 7 µm beads, we applied the same single-band O-PTIR imaging routine used for the 14 µm study. The resulting chemical maps at 1727 cm⁻^1^ are shown in Fig. 2b. As previously described for the 14 µm beads, concentric rings due to the secondary reflection appear on the gold-coated PET and PC filters.

Figure 2c displays the full O-PTIR spectra obtained from three independent 7 µm PMMA beads on each substrate. Individual spectra are shown in Supplementary Fig. S2. On the spectrally silent supports silicon, Anodisc and gold-coated PET, all key PMMA bands (1144, 1270, 1432, 1728, and 2950 cm⁻^1^) appear with flat baselines, yielding mean HQI values of 90, 80, and 79%, respectively. In contrast, spectra collected on gold-coated PC, uncoated PC, cellulose, and silver display show conspicuous substrate features or spike artifacts that depress match quality, with average HQI values of ≈ 58, 50, 55, and 61%, respectively. The gold-coated PC filter deserves separate attention. At 14 µm bead diameter, its performance was comparable to that of a gold-coated PET (HQI ≈ 83%). However, at a 7 µm diameter, its mean HQI decreased sharply to ≈58%. The characteristic PC carbonyl band at 1778 cm^−1^ and the aromatic ring mode at 1504 cm^−1^ were clearly visible in the bead spectra. This size-dependent decrease in spectral match quality can be traced to the coating architecture. The gold-coated PET substrate carries a 100 nm gold layer that is optically opaque to the mid-IR pump beam across the full 934–2992 cm^−1^ range, effectively decoupling the photothermal response of the bead from the underlying polyester. The gold-coated PC substrate, by contrast, has only a ≈ 40 nm gold layer on the receiving side. At 14 µm bead diameter, the photothermal signal is dominated by the bead volume, and the thinner coating is sufficient. For a 7 µm bead, the observed deterioration is consistent with incomplete attenuation of the IR excitation by the thinner gold layer (40 nm), although additional contributions from coating morphology or thermal effects cannot be excluded. A fraction of the excitation reaches the PC base, resulting in the appearance of its characteristic bands in the O-PTIR spectrum. Figure 2d plots the individual HQI scores for all three beads on each substrate. These data confirm that, at the 7 µm scale, reliable PMMA identification is confined to silicon, Anodisc, and gold-coated PET, the only substrates whose spectra remain essentially featureless across the PMMA fingerprint region.

### Raman analysis of 14 µm and 7 µm PMMA beads

**Fig. 3:**
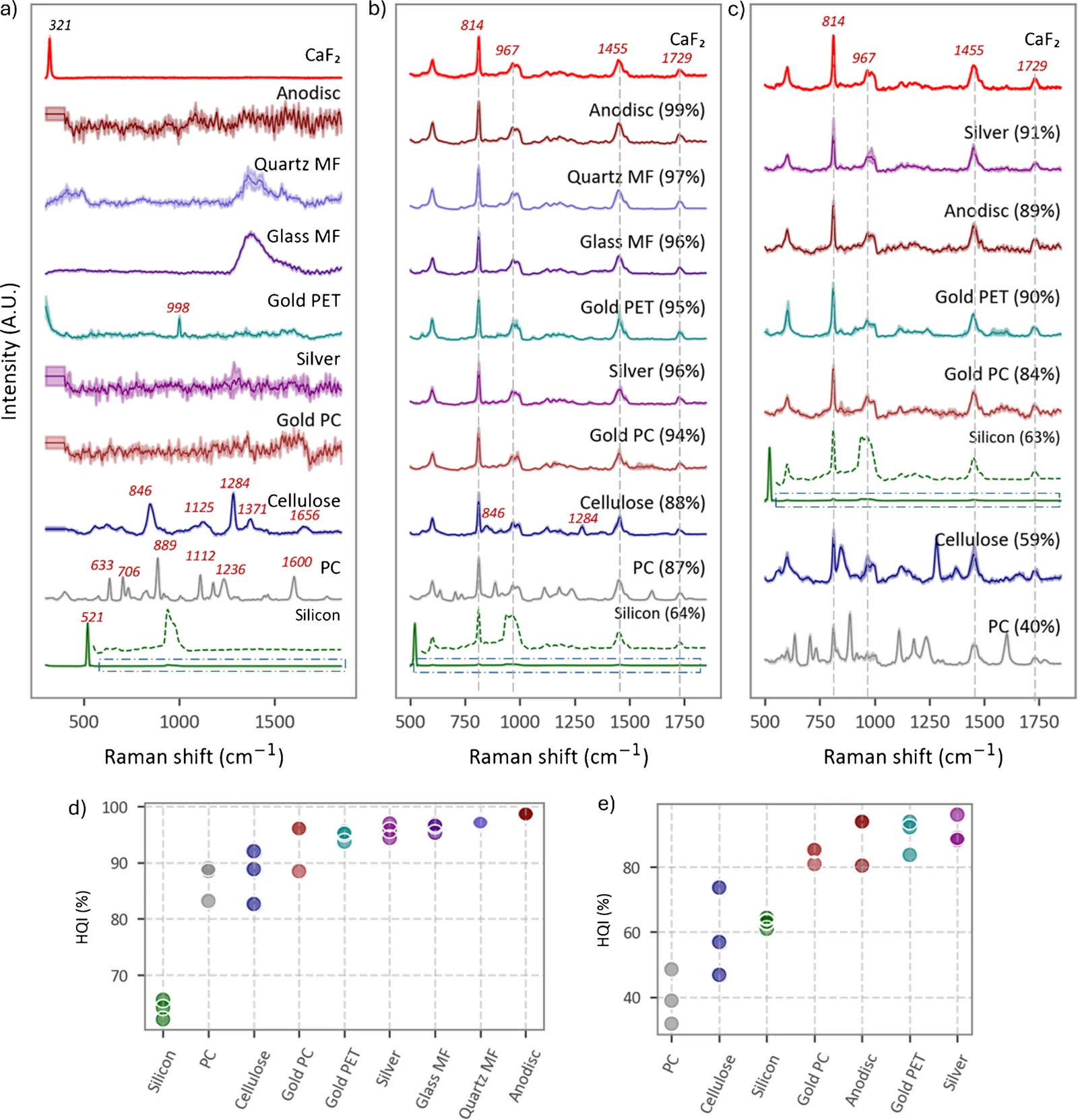
Raman performance of filter substrates with 14 µm and 7 µm PMMA beads. **a** Substrate only Raman spectra. CaF₂ shows its lattice line at 321 cm⁻^1^; silicon displays the 521 cm⁻^1^ phonon band and 958 cm⁻^1^ overtone; glass and quartz microfiber filters add a broad photoluminescence hump (~ 1400 cm⁻^1^); cellulose and PC exhibit their characteristic polymer bands; Anodisc, silver, and gold-coated PC are effectively Raman silent; gold-coated PET retains one PET band at 998 cm⁻^1^. **b** Raman spectra of single 14 µm PMMA beads on each substrate. Substrate bands from cellulose and PC distort the PMMA pattern; silicon’s overtone at 958 cm⁻^1^ also intrudes. **c** Same as **b** for 7 µm beads on the seven substrates where localization was possible. **d** HQI versus a CaF₂ supported PMMA reference for 14 µm beads: Gold-coated PET/PC, silver, Anodisc, and both microfiber filters score 94–99%; cellulose and PC, 88% and 87%; silicon, 64%. **e** HQI distribution for 7 µm beads: silver, gold-coated PET/PC, Anodisc remain ≥ 84%; silicon, cellulose, and PC drop to 63%, 59%, and 53%, respectively, reflecting lattice interference (Si) or substrate fluorescence/overlap (cellulose, PC). Spectra are offset vertically and individually scaled for clarity

Figure 3a shows the Raman background spectra of the nine substrates. The CaF_2_ slide, included as a reference, contributes only its characteristic lattice line at 321 cm⁻^1^. Silicon displays the sharp first order Si phonon band at 521 cm⁻^1^ and a weak second-order overtone near 958 cm⁻^1^. Both glass and quartz microfiber filters are otherwise featureless but generate a broad photoluminescence hump centered around 1400 cm⁻^1^. In contrast, Anodisc, silver, and gold-coated PC filter substrates are effectively Raman silent, offering flat baselines with no detectable lines or fluorescence. The gold-coated PET filter retains a single PET ring breathing band at 998 cm⁻^1^, but is otherwise as clean as silicon and Anodisc in the PMMA fingerprint window. The mixed cellulose ester filter exhibits a cluster of nitro/ester and ring modes at 846, 1125, 1284, 1371, and 1656 cm⁻^1^, whereas polycarbonate displays its characteristic aromatic and carbonate bands at 633, 706, 889, 1112, 1236, and 1600 cm⁻^1^. Figure 3b overlays the Raman spectra of 14 µm PMMA beads on each substrate, with the CaF₂ reference spectrum showing the characteristic PMMA Raman bands at 814, 967, 1455, and 1729 cm⁻^1^. As expected, the cellulose and PC filters contribute their own bands nitro/ester modes at 1284, 1371, and 1656 cm⁻^1^ for cellulose, and aromatic + carbonate bands at 633, 706, 889, 1112, 1236, and 1600 cm⁻^1^ for PC which distort the PMMA pattern and depress the mean HQI to 88% and 87%, respectively. Silicon performs worst (HQI ≈ 64%); even after the 521 cm⁻^1^ phonon band is excluded from the match window, the strong second-order overtone at ~ 958 cm⁻^1^ lowers spectral fidelity. Apart from silicon, cellulose, and PC, all other substrates deliver HQI ≥ 94%, indicating reliable Raman identification of 14 µm PMMA beads. The distribution of individual HQI scores is plotted in Fig. 3d.

Figure 3c shows that, despite the smaller 7 µm bead, four of the seven substrates still return strong matches. Silver, gold-coated PET/PC, and Anodisc each give HQI values of ≥ 84%, maintaining a clear PMMA fingerprint. By contrast, the cellulose filter (59%), PC (53%), and silicon (63%) all fall well below the 75% reliability threshold. The first two do so because their polymer bands overwhelm the spectrum. Silicon falls below the threshold because its strong 521 cm⁻^1^ phonon band and 958 cm⁻^1^ overtone overlap with the PMMA backbone region. Figure 3e plots the individual HQIs on each substrate.

Overall, silver, gold-coated PET/PC, and Anodisc are the most reliable Raman substrates delivering high HQIs for both 14 µm and 7 µm beads. In contrast, silicon, cellulose, and uncoated PC consistently underperform because their intrinsic lattice bands or polymer bands overlap with the PMMA fingerprint region. Notably, bead-to-bead reproducibility is markedly higher in the Raman channel than in the O-PTIR channel (see Figs. 1d, 2c), as reflected by the considerably narrower standard deviation envelopes at both particle sizes. Individual Raman spectra of all beads are shown in Supplementary Fig. S3 for 14 µm diameter and in Fig. S4 for 7 µm. The origin of this difference is addressed in the next section.

## Discussion

Based on the O-PTIR measurements, identification of microplastics broadly requires substrates that meet two conditions: absence of substrate mid-IR absorption bands and a smooth substrate surface that reflects the 785 nm probe beam cleanly. Silicon and Anodisc meet both conditions and gave the highest match scores (HQI ≈ 95%) at 14 µm. Both substrates show minimal mid-IR absorption and provide smooth surfaces for probe reflection. Gold-coated PET/PC, uncoated PC, glass, and quartz microfiber filters also performed well at this particle size (HQI≈80–85%), as their mid-IR bands do not strongly interfere with PMMA identification. Though spectrally silent, silver showed a reduced spectral match (HQI ≈ 77%), likely due to its coarse sintered surface. As seen in a similar LDIR study [19], textured silver degraded image quality even for particles above 20 µm in size. The underlying reason is the O-PTIR contrast mechanism itself. The mid-IR pump beam induces local thermal expansion and a change in the local refractive index, which the 785 nm probe laser detects in reflection. Therefore, surface roughness at the point of interaction affects both the mid-IR excitation and the reflected probe light. Consequently, substrate texture can introduce localized signal variations that degrade spectral quality. However, surface topography was not measured in this study. Cellulose has intrinsic nitro/ester bands that overlap the PMMA fingerprint, lowering its HQI to 72%. At 7 µm, HQI values drop across most substrates, and the effect is most pronounced for substrates with their own mid-IR bands, where the substrate spectral contribution becomes more apparent at the smaller particle size. Only silicon (90%), Anodisc (80%), and gold-coated PET (79%) stayed near or above 80%, while the rest dropped below 62%. The gold-coated filters show an additional effect of coating thickness. Gold-coated PET has a 100 nm layer that fully blocks the mid-IR pump at both bead sizes (HQI ≈ 79%), but the 40 nm layer on gold-coated PC does not fully block the pump at 7 µm, so polycarbonate bands reappear and the HQI drops to 58% (Table 2). Furthermore, careful inspection of the O-PTIR spectra of 14 µm PMMA beads on gold-coated filters revealed that the ratios of the most intense bands at 1728 and 1144 cm^−1^ are smaller than those of other substrates, and the 1728 cm^−1^ band broadens with a high wavenumber shoulder. These changes may be due to the reflection of the IR pump beam on the gold surfaces which can cause standing wave effects within the 14 µm PMMA beads. The size dependence is consistent with the absence of this effect in the 7 µm beads.

**Table 2:**
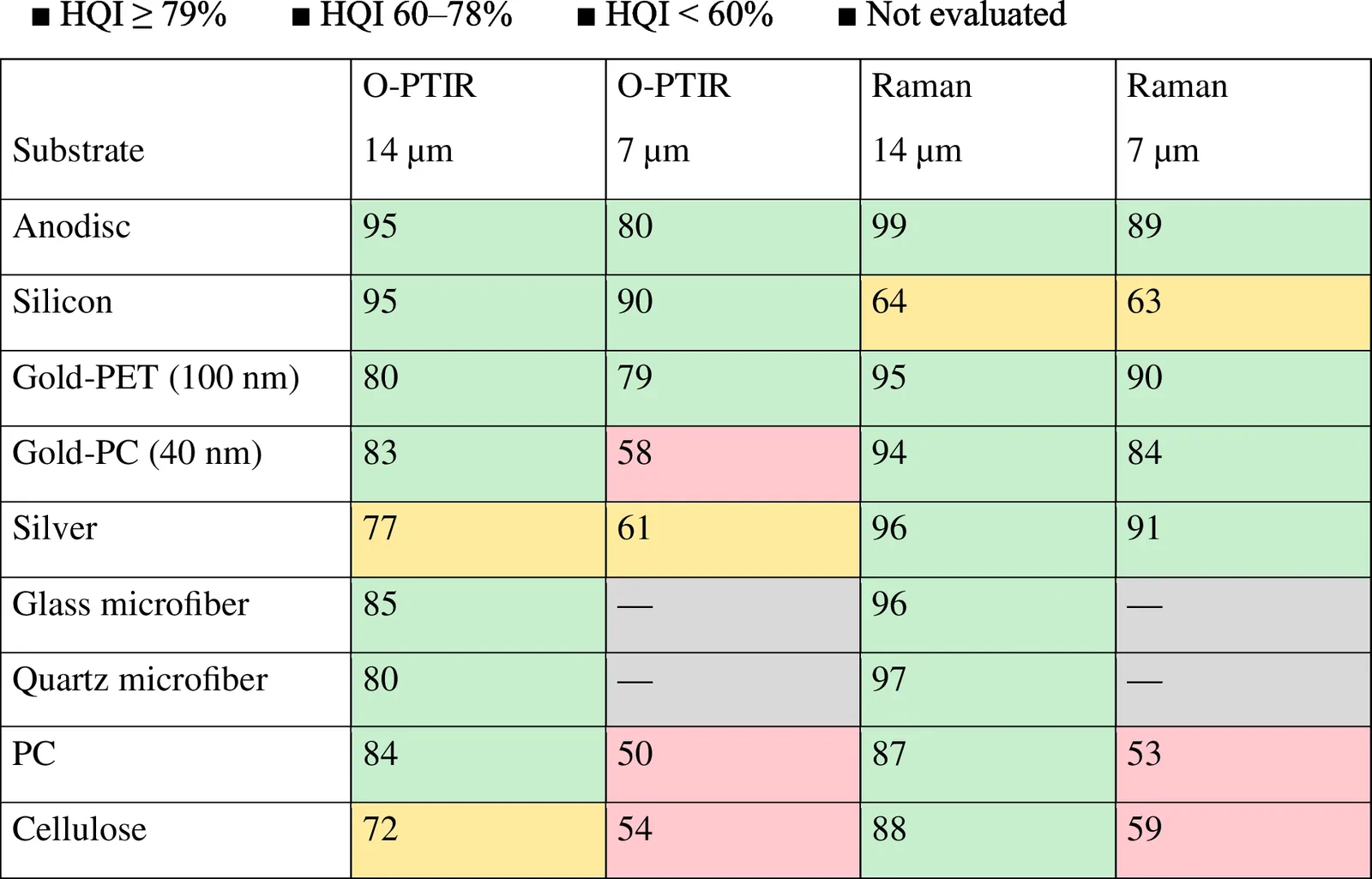
Substrate suitability matrix for O-PTIR and Raman-based microplastic analysis. Cells show approximate mean HQI (%) for 7 and 14 µm PMMA microspheres (*n* = 3). Color coding indicates spectral match quality

In the Raman channel, the requirements differ. What matters is the absence of Raman-active bands from the substrate. Silver, gold-coated PET/PC, and Anodisc meet this criterion, maintaining HQI ≥ 94% for 14 µm beads and ≥ 84% for 7 µm beads. The polymeric filters (PC and cellulose) introduce their own Raman bands, though at 14 µm, the bead signal still dominates (HQI ≈ 87–88%). At 7 µm, their substrate bands overwhelm the weaker bead signal, dropping HQI to ≈ 53–59%. Silicon, despite being the top performer O-PTIR substrate, performs poorly in the Raman channel at both particle sizes. Its intense first-order phonon band at 521 cm⁻^1^ and a second-order overtone near 958 cm⁻^1^ overlap with the PMMA region, depressing the HQI to ≈ 64% and 63% for 14 µm and 7 µm beads respectively, even after the 521 cm⁻^1^ band is excluded from the match window. It seems that the range around 958 cm^−1^ needs to be excluded as well.

Beyond substrate-specific spectral interference, the two channels also differ markedly in measurement reproducibility. Across all substrates and both bead sizes, the O-PTIR standard deviation envelopes are considerably wider than those of the corresponding Raman spectra (cf. Figures 1d, 2c vs. 3b, c). This asymmetry is rooted in the fundamentally different detection mechanisms of the two modalities. In O-PTIR, the pulsed QCL pump beam heats the bead, and then the visible probe detects the resulting refractive index and light scattering modulation. The amplitude of this photothermal signal depends not only on the mid-IR absorbance but also on how rapidly the absorbed heat dissipates, a process governed by the substrate’s thermal conductivity and the particle’s thermal diffusion length [29, 30]. For example, COMSOL simulations show that the thermal decay time of a 500 nm PMMA particle drops by a factor of roughly 3.5 when a silicon substrate is introduced beneath the particle in place of a free-standing air environment [30], illustrating how sensitively the photothermal response tracks the local thermal environment. On a real filter surface, the contact geometry between a spherical bead and the substrate varies with the bead’s precise landing position flat region, pore edge, or a surface ridge. Replicate beads of identical composition therefore experience different thermal coupling and yield different O-PTIR signal amplitudes. Raman scattering, by contrast, is generated throughout the laser illuminated volume of the bead via an instantaneous, non-thermal process; its intensity is governed by the molecular polarizability and the excitation power, not by how heat flows into the substrate. The signal is therefore insensitive to the bead substrate thermal interface, consistent with the narrow standard deviation envelopes observed in Fig. 3b and c. Table 2 summarizes the substrate suitability across both modalities and particle sizes.

In the microplastics spectroscopy literature, an HQI threshold of 0.70 is the most widely applied cutoff for polymer identification, though this value was established by precedent rather than systematic validation [31, 32]. Recent automated Raman work on submicron particles reports median HQI values exceeding 79% for all tested polymer types on optimized substrates, indicating that values in this range are sufficient for reliable identification [33]. On this basis, we consider substrates yielding HQI ≥ 79% suitable for polymer identification in both the O-PTIR and Raman channels. Based on these findings, the present data support practical recommendations for substrate selection. For laboratories operating combined O-PTIR/Raman workflows, Anodisc and gold-coated PET (100 nm, single-sided) emerge as the strongest compromise substrates. Both maintain O-PTIR HQIs ≥ 79% and Raman HQIs ≥ 84% at the 7 µm scale. Where only O-PTIR is required, silicon offers the cleanest baseline and highest HQI overall. For Raman only applications, silver and gold-coated PET/PC provide the best spectral fidelity. Uncoated polycarbonate and mixed cellulose ester filters should be avoided for either modality, as their intrinsic polymer bands overlap the fingerprint region of most common microplastics. In addition to spectral suitability, the substrates differ in terms of practical handling. In our experience, the gold-coated PET and PC filters, as well as the silicon, PC, and cellulose filters, were robust and easy to handle. However, the silver filter is prone to scratches from tweezers during handling. The Anodisc filter is brittle, though its plastic support ring makes it easier to handle. While the glass and quartz microfiber filters were easy to handle, the low optical contrast of their fiber mats complicates particle localization. Additionally, small particles may become embedded between the fibers, which would hinder their recovery and detection in samples. The extent of the substrate’s influence also depends on the particles’ optical properties. Although the mid-IR pump radiation is largely absorbed within the bead, the substrate still affects the O-PTIR signal in three ways. First, PMMA is transparent at 785 nm, so the probe light is reflected or backscattered at the substrate, as evidenced by the interference rings on the gold-coated filters (see Figs. 1b, 2b). Second, the photothermal signal depends on the thermal contact at the bead-filter interface. Third, the IR pump beam cannot be focused below its mid-IR wavelength, so it partially illuminates the exposed substrate at the bead perimeter. This contributes to stronger substrate bands for the smaller beads. For opaque or strongly scattering particles, such as pigmented environmental microplastics, the first interaction is expected to be weaker and remains to be investigated.

An important thing to note is that PMMA was used as the test polymer because it is readily available as microspheres in defined sizes, which allowed controlled and reproducible measurement conditions across all substrates. Standardized microspheres of known size (7 µm and 14 µm) and uniform surface condition were chosen so that differences in spectral quality can be attributed to the substrate rather than to particle shape or surface variability. The results reported here were obtained using single-point measurements, in which the probe was placed in the optimal position on each bead. Automated particle finding or hyperspectral imaging would encounter additional filter-related issues. For example, particles may be difficult to see on certain substrates, or the laser may miss the center of the particle. Microscopy options, such as darkfield contrast [33] and cross-polarization, have already been introduced for improved particle location. Therefore, the substrate ranking may differ for these methods. Future studies with other microplastics, such as PE and PP, and irregularly shaped environmental particles would further strengthen the substrate recommendations for microplastic analysis.

## Conclusions

This study demonstrates that no single filter substrate is optimal for both O-PTIR and Raman microplastic analysis, because each technique imposes different requirements on the substrate. O-PTIR demands both mid-IR spectral neutrality and surface smoothness, whereas Raman performance depends primarily on the absence of substrate Raman bands within the polymer fingerprint region. As a result, substrate rankings differ between the two techniques, and recommendations from conventional FTIR or Raman studies cannot be applied directly to O-PTIR. Among the nine substrates tested, Anodisc and gold-coated PET offer the most consistent performance across both channels and both particle sizes. The divergent performance of 40 nm and 100 nm gold coatings shows that coating thickness is a determining factor for substrate suitability at small particle sizes. Future work should systematically evaluate a range of gold coating thicknesses to establish the minimum layer thickness required for effective mid-IR attenuation across different particle sizes. Aluminum-coated PET filters with comparable properties are available as a lower cost variant of gold-coated PET filters. Silicon gives high-quality O-PTIR spectra because the polished surface efficiently reflects the visible probe light. The lower HQI of PMMA on silicon with Raman can be improved by excluding the overtone range around 958 cm^−1^. Further studies with other microplastic types, environmental samples, and automated or imaging-based workflows will show how far these recommendations can be generalized. By providing the first systematic substrate comparison for O-PTIR spectroscopy, this work offers a practical foundation for filter selection in multimodal microplastic analysis.

## Supplementary Information

Below is the link to the electronic supplementary material.

Supplementary file1 (DOCX 3.06 MB)

## Data Availability

Data will be made available on reasonable request.
