# Supplementary material for: Evaluation of filter substrates for concurrent optical photothermal infrared and Raman spectroscopy in microplastic analysis
Source: Anal Bioanal Chem. 2026 Aug 25;418(22):7409–20. doi: 10.1007/s00216-026-06735-4 (PMC13736340; doi:10.1007/s00216-026-06735-4)
Supplement: Supplementary file 1 [file 216_2026_6735_MOESM1_ESM.docx]

**Supplementary Information**

**Evaluation of Filter Substrates for Concurrent Optical Photothermal Infrared and Raman Spectroscopy in Microplastic Analysis**

Shiwani Shiwani^1^, Shravan Raghunathan^1^, Jürgen Popp^1,2^, Iwan W. Schie^1,3^, Christoph Krafft^1,*^

^1^ Leibniz Institute of Photonic Technology, Albert-Einstein-Straße 9, 07745 Jena, Germany

^2^ Institute of Physical Chemistry and Abbe Center of Photonics, Friedrich Schiller University Jena, Helmholtzweg 4, 07743 Jena, Germany

^3^ Department for Medical Engineering and Biotechnology, University of Applied Sciences Jena, Carl-Zeiss-Promenade 2, 07745 Jena, Germany

* Corresponding author: christoph.krafft@leibniz-ipht.de

**Fig. S1** Individual area-normalized O-PTIR spectra of the three 14 µm PMMA beads per substrate used in Fig. 1, together with the CaF_2_ supported reference beads.

**Fig. S2** Individual area-normalized O-PTIR spectra of the three 7 µm PMMA beads per substrate used in Fig. 2, together with the CaF_2_ supported reference beads. Glass and quartz microfiber filters were excluded at this particle size because the low optical contrast of their fiber mats prevented reliable bead localization (see main text).

**Fig. S3** Individual area-normalized Raman spectra of the three 14 µm PMMA beads per substrate used in Fig. 3b, together with the CaF_2_ supported reference beads. Spectra were baseline corrected as described in the Methods. The silicon panel is shown above 530 cm^-1^ to exclude the dominant Si phonon band at 521 cm^-1^.

**Fig. S4** Individual area-normalized Raman spectra of the three 7 µm PMMA beads per substrate used in Fig. 3c, together with the CaF_2_ supported reference beads. Spectra were baseline corrected as described in the Methods. The silicon panel is shown above 530 cm^-1^ to exclude the dominant Si phonon band at 521 cm^-1^. Glass and quartz microfiber filters were excluded at this particle size (see main text).
